# Supplementary material for: Probabilistic modeling methods for cell-free DNA methylation based cancer classification
Source: BMC Bioinformatics. 2022 Apr 4;23:119. doi: 10.1186/s12859-022-04651-9 (PMC8978416; doi:10.1186/s12859-022-04651-9)
Supplement: Supplementary file 1 — Additional file 1. Supplementary figures and tables. [file 12859_2022_4651_MOESM1_ESM.pdf]

Probabilistic modeling methods for cell-free DNA methylation based cancer classification

Supplementary Information

Viivi Halla-aho and Harri Lähdesmäki

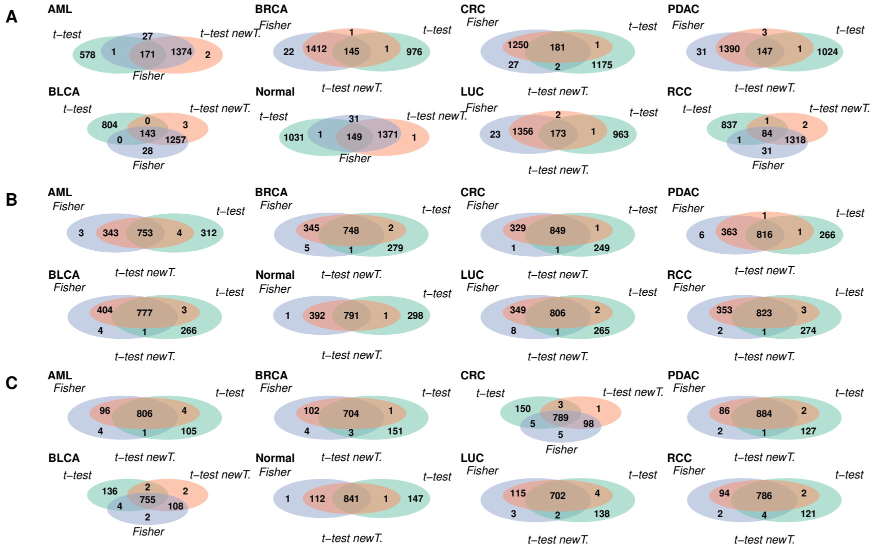

Figure S1: Number of overlapping DMRs between the DMRs found with Fisher's exact test, moderated t-test with the original data transformation and moderated t-test with new data transformation. DMRs from the 100 data splits were combined and only the DMRs present in the DMR set of at least 50 data splits were kept for finding the overlaps. The results are presented for thinning with total read counts 10^4, 10^5 and 10^6 in figures **A**, **B** and **C**, respectively.

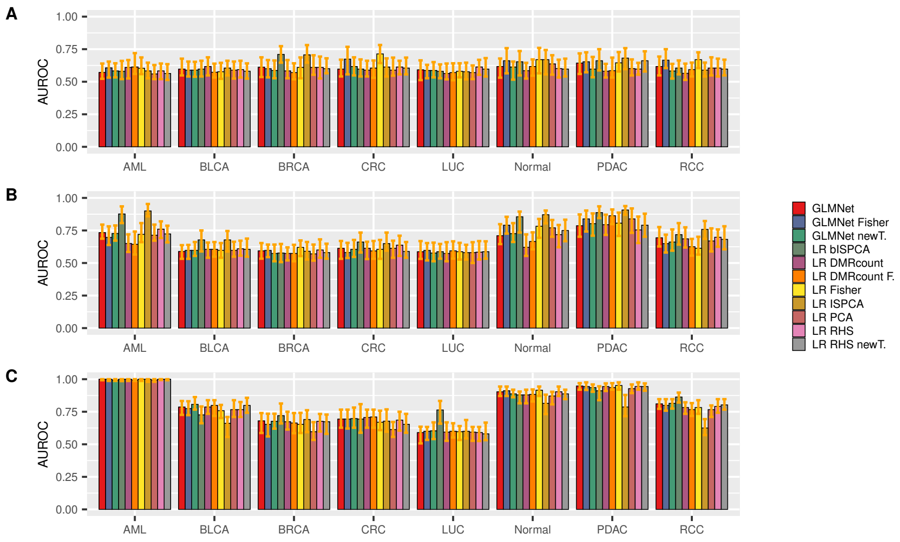

Figure S2: Barplots of the AUROC medians over the 100 data splits for all approaches. The orange bar on top of each bar shows the 25% and 75% quantiles. The AUROC values have been calculated for the test data sets in the discovery cohort. The results are presented for each class separately. The results are presented for thinning with total read counts 10^4, 10^5 and 10^6 in figures **A**, **B** and **C**, respectively.

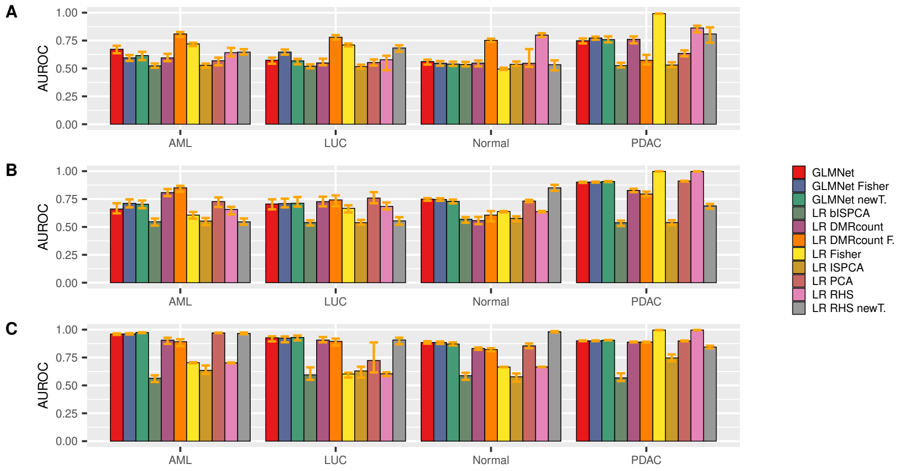

Figure S3: Barplot of the AUROC medians over the 100 data splits for all approaches on the validation cohort. The orange bar on top of each bar shows the 25% and 75% quantiles. The results are presented for each class separately. The results are presented for thinning with total read counts 10^4, 10^5 and 10^6 in figures **A**, **B** and **C**, respectively.

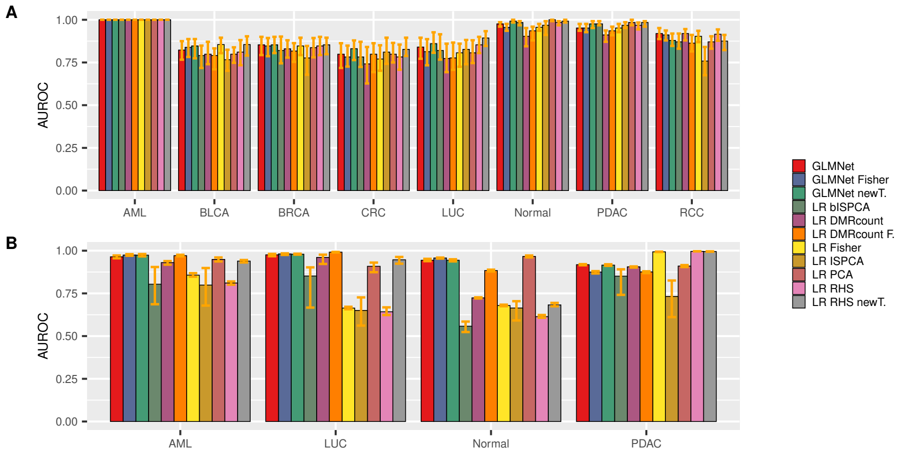

Figure S4: The bars represent the AUROC medians over the 100 data splits for the non-thinned data set for each of the methods. The orange bar on top of each bar shows the AUROC range from 25% quantile to 75% quantile. **A**: AUROC medians for discovery cohort. **B**: AUROC medians for validation cohort.

Table S1: Medians of AUROC and AUPRC values over 100 data splits for discovery cohort, subsampled data with total read count 10^4. Highest values have been bolded.

		LR RHS	GLMNet	LR PCA	LR Fisher	LR DMRcount	LR DMRcount F.	LR ISPCA	LR bISPCA	LR RHS newT.	GLMNet newT.	GLMNet Fisher
AML	AUROC	0.583	0.572	0.560	0.603	0.610	**0.613**	0.583	0.580	0.563	0.583	0.607
	AUPRC	0.149	0.150	0.138	0.130	0.120	**0.263**	0.159	0.155	0.138	0.158	0.160
BLCA	AUROC	0.593	0.597	0.589	0.577	**0.617**	0.573	0.605	0.597	0.581	0.589	0.589
	AUPRC	**0.157**	0.145	0.108	0.120	0.120	0.122	0.116	0.121	0.105	0.094	0.142
BRCA	AUROC	0.610	0.612	0.610	0.610	0.583	0.570	0.707	**0.710**	0.600	0.587	0.600
	AUPRC	0.197	0.188	0.142	0.188	0.159	0.158	0.271	**0.280**	0.160	0.159	0.190
CRC	AUROC	0.613	0.597	0.589	**0.714**	0.589	0.605	0.613	0.597	0.605	0.617	0.673
	AUPRC	0.150	0.127	0.103	0.247	0.111	0.128	0.150	0.154	0.096	0.093	**0.279**
LUC	AUROC	**0.613**	0.592	0.570	0.580	0.563	0.570	0.577	0.580	0.597	0.587	0.587
	AUPRC	0.120	0.134	0.132	0.160	0.141	0.145	**0.192**	0.172	0.128	0.142	0.153
Normal	AUROC	0.601	0.617	0.637	**0.669**	0.585	0.617	**0.669**	0.653	0.597	0.613	0.661
	AUPRC	0.107	0.113	0.098	**0.237**	0.109	0.120	0.199	0.185	0.119	0.111	0.225
PDAC	AUROC	0.597	0.643	0.601	0.645	0.581	0.585	**0.681**	0.661	0.661	0.597	0.653
	AUPRC	0.129	0.158	0.110	**0.243**	0.125	0.105	0.239	0.235	0.191	0.139	0.227
RCC	AUROC	0.605	0.617	0.605	**0.669**	0.565	0.593	0.589	0.605	0.597	0.583	0.665
	AUPRC	0.102	0.099	0.122	**0.185**	0.108	0.114	0.123	0.124	0.107	0.113	**0.185**

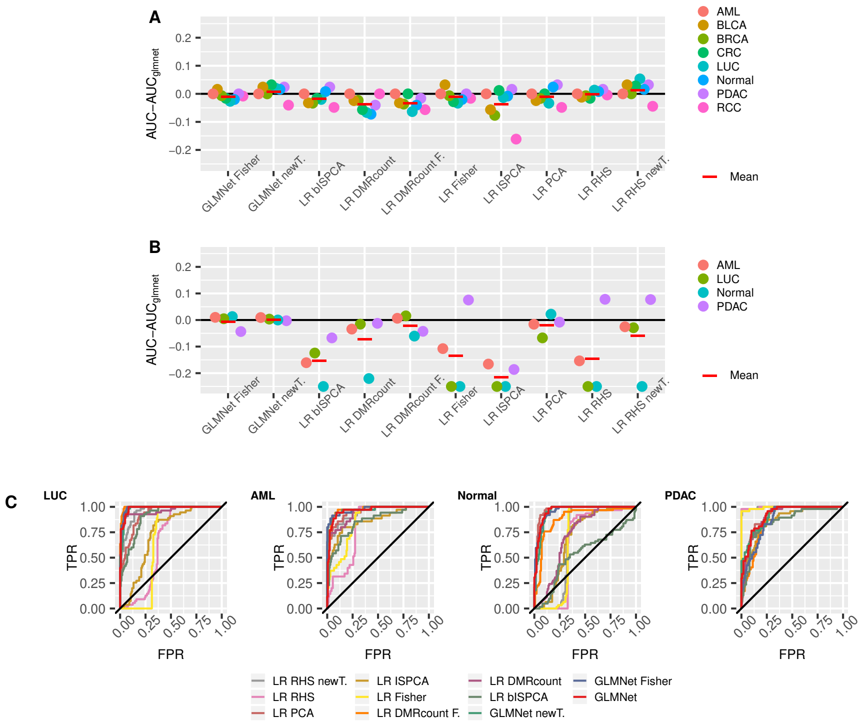

Figure S5: Assessing the performance of the different classifiers for the non-thinned data. **A**: The median AUROC differences between the original GLMnet method and the other classifiers for the discovery cohort test sets. The median has been calculated over the 100 data splits. **B**: The median AUROC differences between the original GLMnet method and the other classifiers for the validation cohort data. The median has been calculated over the 100 data splits. The negative values have been truncated to -0.25. **C**: Validation cohort ROCs calculated with prediction means over 100 data splits and corresponding fitted models. The results are presented for each class separately. Red lines indicate the means over the four classes.

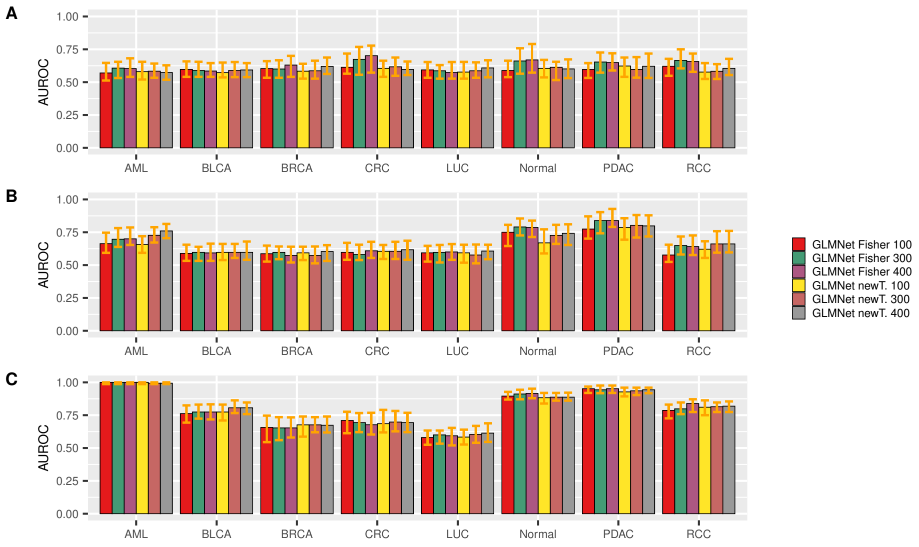

Figure S6: Comparison of different DMR numbers. AUROC barplots for the discovery cohort. The orange bar on top of each bar shows the 25% and 75% quantiles. The results are presented for thinning with total read counts 10^4, 10^5 and 10^6 in figures **A**, **B** and **C**, respectively.

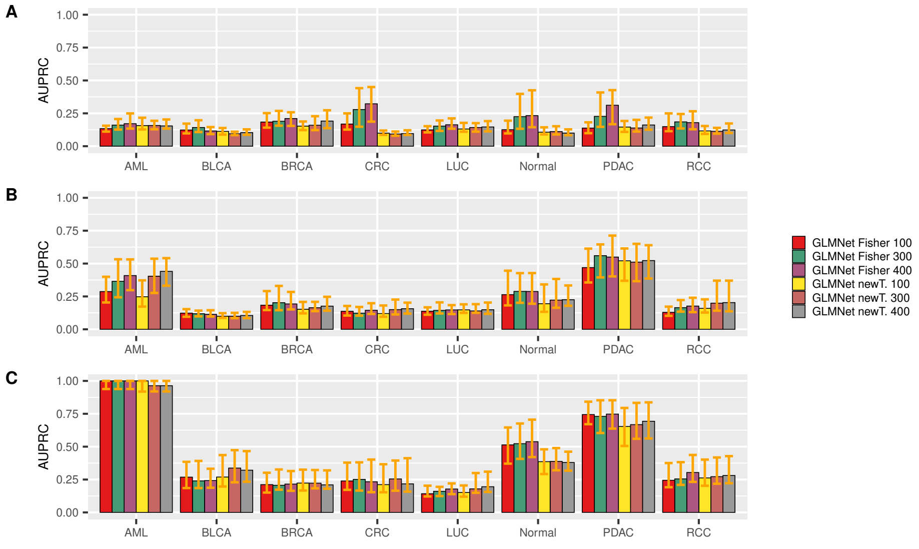

Figure S7: Comparison of different DMR numbers. AUPRC barplot for the discovery cohort. The orange bar on top of each bar shows the 25% and 75% quantiles. The results are presented for thinning with total read counts 10^4, 10^5 and 10^6 in figures **A**, **B** and **C**, respectively.

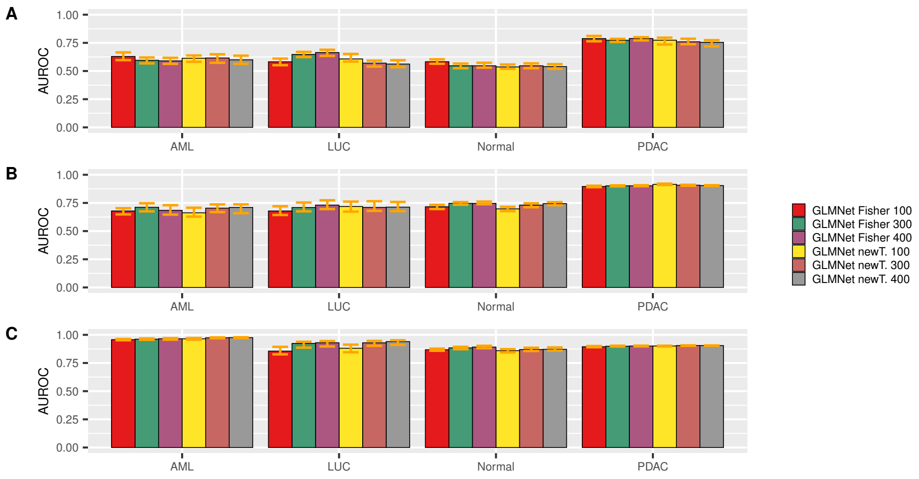

Figure S8: Comparison of different DMR numbers. AUROC barplot for the validation cohort. The orange bar on top of each bar shows the 25% and 75% quantiles. The results are presented for thinning with total read counts 10^4, 10^5 and 10^6 in figures **A**, **B** and **C**, respectively.

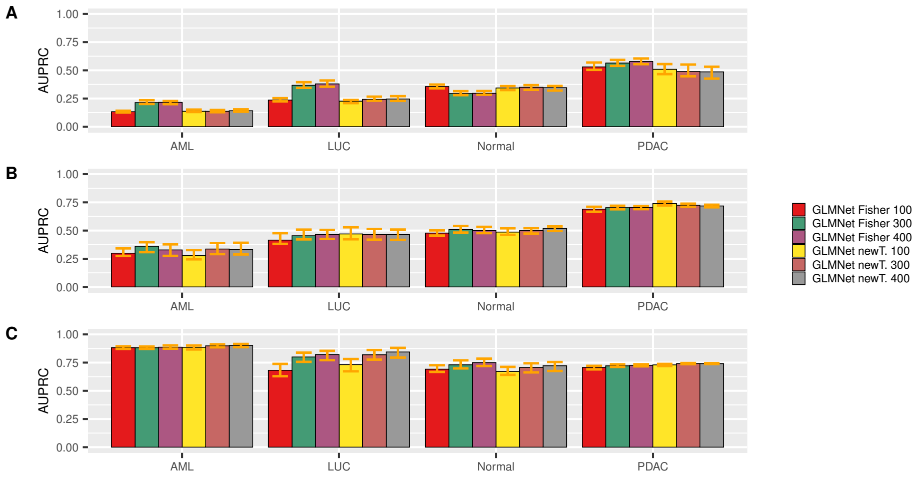

Figure S9: Comparison of different DMR numbers. AUPRC barplot for the validation cohort. The orange bar on top of each bar shows the 25% and 75% quantiles. The results are presented for thinning with total read counts 10^4, 10^5 and 10^6 in figures **A**, **B** and **C**, respectively.

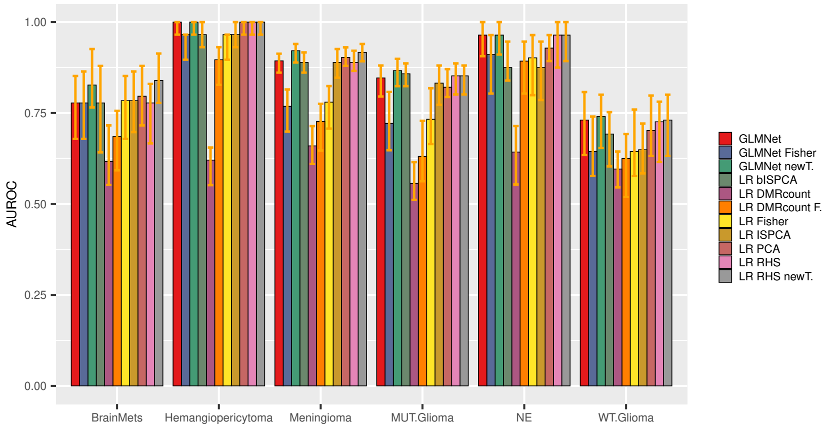

Figure S10: he bars represent the AUROC medians over the 100 data splits for the intracranial tumors data set for each of the methods. The orange bar on top of each bar shows the AUROC range from 25% quantile to 75% quantile. Abbreviations of the class names: BrainMets = brain metastases, MUT.Glioma= IDH mutant glioma, NE= low-grade glioneuronal and WT.Glioma= IDH wildtype glioma.

Table S2: Medians of AUROC and AUPRC values over 100 data splits for discovery cohort, subsampled data with total read count 10^5. Highest values have been bolded.

		LR RHS	GLMNet	LR PCA	LR Fisher	LR DMRcount	LR DMRcount F.	LR lSPCA	LR bISPCA	LR RHS newT.	GLMNet newT.	GLMNet Fisher
AML	AUROC	0.760	0.733	0.713	0.720	0.650	0.643	**0.900**	0.877	0.723	0.727	0.697
	AUPRC	0.470	0.422	0.394	0.412	0.233	0.222	**0.703**	0.699	0.379	0.404	0.365
BLCA	AUROC	0.609	0.589	0.597	0.597	0.605	0.605	**0.677**	**0.677**	0.605	0.597	0.597
	AUPRC	0.092	0.104	0.102	0.113	0.113	0.127	**0.191**	0.174	0.106	0.100	0.119
BRCA	AUROC	0.600	0.593	0.570	**0.620**	0.573	0.573	0.590	0.577	0.580	0.573	0.597
	AUPRC	0.211	0.198	0.166	0.220	0.158	0.144	0.175	0.176	0.168	0.164	0.202
CRC	AUROC	0.637	0.613	0.613	0.605	0.613	0.593	0.649	**0.661**	0.589	0.605	0.581
	AUPRC	0.161	0.145	0.152	0.133	0.126	0.121	**0.284**	0.260	0.137	0.150	0.123
LUC	AUROC	0.583	0.587	0.580	0.587	0.573	0.593	0.580	0.587	0.587	0.577	**0.597**
	AUPRC	0.141	0.147	0.139	0.146	0.152	**0.160**	0.157	0.151	0.129	0.140	0.144
Normal	AUROC	0.718	0.710	0.770	0.782	0.621	0.665	**0.871**	0.855	0.75	0.726	0.79
	AUPRC	0.210	0.219	0.250	0.318	0.163	0.177	**0.337**	0.307	0.256	0.222	0.288
PDAC	AUROC	0.754	0.786	0.839	0.806	0.794	0.863	**0.907**	0.887	0.79	0.802	0.839
	AUPRC	0.446	0.490	0.566	0.502	0.540	0.610	**0.643**	0.630	0.531	0.511	0.560
RCC	AUROC	0.698	0.694	0.669	0.613	0.685	0.625	**0.758**	0.718	0.681	0.661	0.649
	AUPRC	0.206	0.201	0.210	0.156	0.215	0.152	**0.238**	0.230	0.234	0.199	0.165

Table S3: Medians of AUROC and AUPRC values over 100 data splits for discovery cohort, subsampled data with total read count 10^6. Highest values have been bolded.

		LR RHS	GLMNet	LR PCA	LR Fisher	LR DMRcount	LR DMRcount F.	LR ISPCA	LR bISPCA	LR RHS newT.	GLMNet newT.	GLMNet Fisher
AML	AUROC	**1.000**	**1.000**	0.993	**1.000**	**1.000**	0.993	**1.000**	**1.000**	**1.000**	0.993	**1.000**
	AUPRC	**1.000**	**1.000**	0.964	**1.000**	**1.000**	0.964	**1.000**	**1.000**	**1.000**	0.964	**1.000**
BLCA	AUROC	0.766	0.786	0.766	0.758	0.786	0.798	0.661	0.726	0.798	**0.806**	0.774
	AUPRC	0.253	0.253	0.237	0.235	0.260	0.262	0.182	0.209	**0.338**	0.337	0.240
BRCA	AUROC	0.677	0.680	0.597	0.653	0.673	0.663	0.690	**0.720**	0.673	0.677	0.653
	AUPRC	0.233	0.224	0.168	0.207	0.227	0.231	**0.309**	0.322	0.239	0.222	0.206
CRC	AUROC	0.685	0.694	0.613	0.669	0.706	**0.710**	0.677	0.694	0.653	0.698	0.694
	AUPRC	0.202	0.223	0.143	0.225	0.231	0.242	0.196	0.383	0.173	**0.254**	0.251
LUC	AUROC	0.590	0.590	0.593	0.597	0.593	0.600	0.600	**0.763**	0.580	0.603	0.600
	AUPRC	0.150	0.157	0.165	0.155	0.171	0.166	0.185	**0.431**	0.160	0.179	0.160
Normal	AUROC	0.903	0.903	0.871	**0.915**	0.879	0.883	0.815	0.879	0.887	0.887	0.911
	AUPRC	0.533	0.522	0.375	**0.544**	0.430	0.474	0.360	0.408	0.372	0.388	0.523
PDAC	AUROC	0.944	**0.948**	0.927	0.952	0.944	0.935	0.786	0.911	0.944	0.935	0.944
	AUPRC	0.736	0.736	0.660	**0.762**	0.733	0.723	0.425	0.667	0.673	0.667	0.730
RCC	AUROC	0.790	0.810	0.766	0.782	0.782	0.766	0.625	**0.863**	0.802	0.815	0.798
	AUPRC	0.277	0.281	0.204	0.249	0.242	0.214	0.147	**0.319**	0.266	0.272	0.255

Table S4: Medians of AUROC and AUPRC values over 100 data splits for validation cohort, subsampled data with total read count 10^4. Highest values have been bolded.

		LR RHS	GLMNet	LR PCA	LR Fisher	LR DMRcount	LR DMRcount F.	LR ISPCA	LR bISPCA	GLMNet newT.	GLMNet Fisher	LR RHS newT.
AML	AUROC	0.641	0.672	0.569	0.720	0.595	**0.809**	0.528	0.523	0.595	0.616	0.646
	AUPRC	0.131	0.126	0.148	0.116	0.137	**0.411**	0.198	0.200	0.214	0.136	0.133
LUC	AUROC	0.578	0.574	0.553	0.710	0.550	**0.780**	0.516	0.520	0.647	0.570	0.683
	AUPRC	0.266	0.244	0.250	0.190	0.254	**0.489**	0.282	0.281	0.368	0.243	0.223
Normal	AUROC	**0.799**	0.561	0.546	0.495	0.548	0.752	0.537	0.536	0.547	0.546	0.534
	AUPRC	**0.598**	0.374	0.374	0.280	0.342	0.207	0.294	0.299	0.294	0.349	0.349
PDAC	AUROC	0.863	0.749	0.635	**0.991**	0.761	0.573	0.531	0.525	0.773	0.759	0.809
	AUPRC	0.144	0.453	0.319	**0.984**	0.529	0.211	0.238	0.237	0.565	0.489	0.406

Table S5: Medians of AUROC and AUPRC values over 100 data splits for validation cohort, subsampled data with total read count 10^5. Highest values have been bolded.

		LR RHS	GLMNet	LR PCA	LR Fisher	LR DMRcount	LR DMRcount F.	LR ISPCA	LR bISPCA	GLMNet newT.	GLMNet Fisher	LR RHS newT.
AML	AUROC	0.657	0.660	0.727	0.606	0.808	**0.850**	0.552	0.547	0.711	0.703	0.546
	AUPRC	0.125	0.296	0.360	0.134	0.482	**0.552**	0.210	0.195	0.361	0.336	0.165
LUC	AUROC	0.684	0.705	**0.759**	0.666	0.726	0.742	0.538	0.539	0.709	0.711	0.554
	AUPRC	0.195	0.452	**0.516**	0.199	0.448	0.445	0.312	0.309	0.454	0.464	0.297
Normal	AUROC	0.637	0.749	0.732	0.638	0.557	0.606	0.577	0.569	0.747	0.73	**0.850**
	AUPRC	0.349	0.511	0.456	0.350	0.277	0.254	0.371	0.362	0.510	0.501	**0.811**
PDAC	AUROC	**0.998**	0.901	0.911	**0.998**	0.827	0.795	0.539	0.538	0.902	0.907	0.688
	AUPRC	0.993	0.699	0.739	**0.994**	0.660	0.652	0.247	0.243	0.703	0.725	0.297

Table S6: Medians of AUROC and AUPRC values over 100 data splits for validation cohort, subsampled data with total read count 10^6. Highest values have been bolded.

		LR RHS	GLMNet	LR PCA	LR Fisher	LR DMRcount	LR DMRcount F.	LR ISPCA	LR bISPCA	GLMNet newT.	GLMNet Fisher	LR RHS newT.
AML	AUROC	0.702	0.960	0.970	0.703	0.905	0.892	0.635	0.564	0.961	**0.974**	0.966
	AUPRC	0.245	0.883	0.875	0.245	0.798	0.747	0.233	0.198	0.882	0.898	**0.906**
LUC	AUROC	0.604	0.927	0.723	0.601	0.906	0.895	0.629	0.594	0.923	**0.929**	0.907
	AUPRC	0.295	0.815	0.271	0.294	0.781	0.742	0.370	0.359	0.800	**0.819**	0.781
Normal	AUROC	0.666	0.884	0.855	0.665	0.830	0.821	0.577	0.587	0.884	0.870	**0.981**
	AUPRC	0.367	0.739	0.675	0.367	0.626	0.605	0.267	0.259	0.730	0.707	**0.954**
PDAC	AUROC	**0.996**	0.900	0.899	**0.996**	0.888	0.888	0.747	0.568	0.899	0.905	0.844
	AUPRC	**0.992**	0.724	0.712	0.991	0.706	0.706	0.525	0.210	0.721	0.742	0.538

Table S7: Medians of AUROC and AUPRC values over 100 data splits for discovery cohort, non-thinned data. Highest values have been bolded.

		LR RHS	GLMNet	LR PCA	LR Fisher	LR DMRcount	LR DMRcount F.	LR ISPCA	LR bISPCA	GLMNet newT.	GLMNet Fisher	LR RHS newT.
AML	AUROC	**1.000**	**1.000**	**1.000**	**1.000**	**1.000**	**1.000**	**1.000**	**1.000**	**1.000**	**1.000**	**1.000**
	AUPRC	**1.000**	**1.000**	**1.000**	**1.000**	**1.000**	**1.000**	**1.000**	**1.000**	**1.000**	**1.000**	**1.000**
BLCA	AUROC	0.810	0.823	0.798	**0.855**	0.798	0.790	0.766	0.790	0.839	0.847	**0.855**
	AUPRC	0.360	0.380	0.282	0.396	0.321	0.225	0.350	0.265	0.323	0.435	**0.447**
BRCA	AUROC	0.847	**0.853**	0.837	0.847	0.830	0.817	0.777	0.820	0.847	**0.853**	**0.853**
	AUPRC	**0.558**	0.521	0.484	0.534	0.493	0.444	0.529	0.549	0.518	0.532	0.547
CRC	AUROC	0.782	0.798	0.798	0.770	0.742	0.798	0.810	0.782	0.782	**0.831**	0.827
	AUPRC	0.582	0.580	0.560	0.585	0.563	0.568	0.580	0.576	0.583	0.587	**0.599**
LUC	AUROC	0.853	0.840	0.807	0.807	0.773	0.777	0.827	0.820	0.813	0.860	**0.893**
	AUPRC	0.594	0.662	0.589	0.507	0.433	0.368	0.570	0.665	0.532	**0.743**	0.735
Normal	AUROC	0.984	0.976	**1.000**	0.956	0.903	0.935	0.968	0.984	0.956	0.992	0.992
	AUPRC	0.909	0.837	**1.000**	0.744	0.561	0.562	0.797	0.872	0.736	0.944	0.944
PDAC	AUROC	0.968	0.952	**0.984**	0.952	0.911	0.935	0.968	0.976	0.952	0.976	**0.984**
	AUPRC	0.842	0.748	**0.909**	0.758	0.541	0.699	0.812	0.872	0.751	0.853	0.884
RCC	AUROC	0.915	0.919	0.871	0.903	**0.919**	0.863	0.758	0.871	0.911	0.879	0.875
	AUPRC	0.477	0.535	0.350	0.477	**0.559**	0.400	0.239	0.373	0.518	0.398	0.335

Table S8: Medians of AUROC and AUPRC values over 100 data splits for validation cohort, non-thinned data. Highest values have been bolded.

		LR RHS	GLMNet	LR PCA	LR Fisher	LR DMRcount	LR DMRcount F.	LR ISPCA	LR bISPCA	GLMNet newT.	GLMNet Fisher	LR RHS newT.
AML	AUROC	0.811	0.964	0.948	0.857	0.930	0.971	0.799	0.804	**0.974**	**0.974**	0.939
	AUPRC	0.402	0.807	0.865	0.581	0.791	0.902	0.577	0.554	**0.938**	0.933	0.843
LUC	AUROC	0.642	0.976	0.909	0.664	0.960	**0.991**	0.650	0.852	0.981	0.979	0.947
	AUPRC	0.316	0.932	0.784	0.329	0.901	**0.976**	0.219	0.669	0.945	0.940	0.869
Normal	AUROC	0.614	0.944	**0.966**	0.679	0.724	0.884	0.664	0.558	0.957	0.944	0.683
	AUPRC	0.342	0.853	**0.911**	0.372	0.453	0.712	0.247	0.351	0.900	0.847	0.374
PDAC	AUROC	**0.996**	0.918	0.910	0.993	0.906	0.875	0.732	0.851	0.875	0.915	0.995
	AUPRC	**0.992**	0.783	0.760	0.986	0.664	0.627	0.478	0.626	0.669	0.803	0.989

Table S9: Medians of the AUROC and AUPRC values over 100 data splits for intracranial tumors data set. Highest values have been bolded.

		LR RHS	GLMNet	LR PCA	LR Fisher	LR DMRcount	LR DMRcount F.	LR ISPCA	LR bISPCA	GLMNet Fisher	GLMNet newT.	LR RHS newT.
Brain metastases	AUROC	0.778	0.778	0.796	0.784	0.617	0.685	0.784	0.778	0.778	0.827	**0.840**
	AUPRC	0.226	0.311	0.273	0.324	0.131	0.186	0.337	0.302	0.348	**0.488**	0.480
Hemangiopericytoma	AUROC	**1.000**	**1.000**	**1.000**	0.966	0.621	0.897	0.966	0.966	0.966	**1.000**	**1.000**
	AUPRC	**1.000**	**1.000**	**1.000**	0.307	0.038	0.137	0.307	0.307	0.307	**1.000**	**1.000**
Meningioma	AUROC	0.889	0.894	0.903	0.780	0.660	0.727	0.889	0.889	0.769	**0.921**	0.917
	AUPRC	0.844	0.859	0.852	0.746	0.665	0.690	0.813	0.830	0.724	**0.874**	0.869
Low-grade glioneuronal	AUROC	**0.964**	**0.964**	0.929	0.902	0.643	0.893	0.875	0.875	0.911	**0.964**	**0.964**
	AUPRC	0.689	**0.712**	0.615	0.451	0.085	0.491	0.234	0.451	0.544	**0.712**	**0.712**
IDH wildtype glioma	AUROC	0.726	0.731	0.702	0.644	0.596	0.625	0.649	0.692	0.644	**0.740**	0.731
	AUPRC	0.262	0.277	0.275	**0.283**	0.137	0.153	0.216	0.203	0.240	0.257	0.257
IDH mutant glioma	AUROC	0.852	0.847	0.821	0.733	0.557	0.631	0.832	0.858	0.722	**0.866**	0.852
	AUPRC	0.626	0.615	0.597	0.524	0.268	0.418	0.588	0.595	0.502	**0.637**	0.627
